# DirectClean: artifact-aware preprocessing and read rescue for Oxford Nanopore direct-cDNA transcriptome sequencing

**DOI:** 10.1093/nargab/lqag132

**Published:** 2026-10-31

**Authors:** Qingxiang Guo, Yangyang Li, Rendong Yang

**Affiliations:** Department of Urology, Northwestern University Feinberg School of Medicine, 303 E Superior St, Chicago, IL 60611, United States; Department of Urology, Northwestern University Feinberg School of Medicine, 303 E Superior St, Chicago, IL 60611, United States; Department of Urology, Northwestern University Feinberg School of Medicine, 303 E Superior St, Chicago, IL 60611, United States; Robert H. Lurie Comprehensive Cancer Center, Northwestern University Feinberg School of Medicine, 303 E Superior St, Chicago, IL 60611, United States

## Abstract

Oxford Nanopore direct-cDNA sequencing enables full-length transcriptome profiling without PCR amplification, but recurrent chimeric artifacts introduced during reverse transcription and library preparation can compromise isoform reconstruction and gene-fusion analysis. These include foldback inversions, aberrant primer configurations, internal adapter concatemers, and homopolymer-mediated template-switching events. Existing preprocessing strategies primarily address primer-based orientation and adapter detection, leaving several direct-cDNA-specific artifact classes unresolved and often discarding reads that still contain usable transcript sequence. We developed DirectClean, an artifact-aware preprocessing and read-rescue framework integrating strand orientation, artifact removal, and junction-level splitting of recoverable chimeric reads. Benchmarking across four direct-cDNA datasets showed consistently higher read and base retention with DirectClean than with Pychopper. In VCaP, DirectClean retained 65.2% of reads compared with 57.6% for Pychopper, supported additional transcripts, and reduced the chimeric read rate from 30.9% in raw data to 16.9%. Semi-synthetic validation demonstrated accurate rescue of adapter-associated and A/T-rich chimeric reads, and matched real-data analyses showed no enrichment of novel transcript models in split-derived reads relative to matched untouched reads. DirectClean provides a reproducible, openly available framework for artifact-aware preprocessing of direct-cDNA transcriptomes and improves the reliability of downstream isoform and fusion analyses.

## Introduction

Oxford Nanopore direct-cDNA sequencing enables full-length transcript profiling without PCR (polymerase chain reaction) amplification, thereby preserving native transcript structure and avoiding amplification bias [1]. However, raw reads require preprocessing prior to downstream analyses such as transcript annotation and gene fusion detection. A key step is strand orientation, as reads derived from either first- or second-strand complementary DNA (cDNA) must be converted to a consistent 5$^{\prime }$-to-3$^{\prime }$ direction. Preprocessing must also address structural artifacts introduced during reverse transcription (RT) and library preparation, including foldback inversions [2–4], adapter concatemers [5], and RT template switching events [6]. Homopolymer-mediated RT template switching is particularly common in direct-cDNA data, occurring when reverse transcriptase dissociates and re-primes at A/T-rich regions [7, 8]. Filtering these artifacts often discards reads that cannot be confidently classified, reducing usable data, especially in PCR-free protocols where each read represents a unique transcript.

Several tools have been developed for direct-cDNA preprocessing. Pychopper, the standard tool from Oxford Nanopore Technologies (ONT), performs strand orientation and rescues chimeras by detecting internal adapter junctions when valid primer pairs are present at read termini (v2.7.10; https://github.com/epi2me-labs/pychopper). However, reads lacking complete or recognizable terminal primers remain unclassified and are discarded, even if internal sequence is recoverable. Restrander improves strand orientation by leveraging polyA/T signals and flags aberrant primer configurations (e.g. TSO–TSO, RTP–RTP), but does not rescue chimeras and removes all flagged or unorientable reads [5]. NanoPrePro optimizes adapter detection for PCR-based cDNA kits but is not designed for or validated on direct-cDNA data [9]. Importantly, no single existing preprocessing workflow integrates strand orientation and sequence rescue with detection of both foldback inversions and homopolymer-mediated RT template switching. In addition, excluded reads are not revisited for recovery.

Here, we present DirectClean, an end-to-end preprocessing pipeline for ONT direct-cDNA data that integrates strand orientation, artifact removal, and sequence rescue. DirectClean incorporates Breakinator for foldback inversion removal [4] and Restrander for strand correction and primer artifact identification [5], and introduces three additional modules. Unknowns rescue recovers orientable reads from initially unclassified sequences. Adapter rescue applies an anchor-and-extend strategy to detect internal adapter junctions, splits reads at the detected junctions, and retains resulting segments that meet the minimum-length requirement. Homopolymer rescue identifies RT template switching at A/T-rich junctions and splits chimeras while retaining flanking sub-reads. By resolving rather than discarding chimeric reads, DirectClean improves data retention. We evaluate rescue performance using semi-synthetic ground-truth reads and matched real-data controls. Across four direct-cDNA datasets, DirectClean retains more reads and bases than Pychopper. On VCaP prostate cancer direct-cDNA data, it reduces chimeric read rates from 30.9% to 16.9% and provides read support for additional transcripts relative to Pychopper.

## Methods

### Pipeline overview

DirectClean processes ONT direct-cDNA FASTQ reads through five stages (Fig. 1a). Stage 1 applies Breakinator [4] to remove foldback inversions. Stage 2 uses Restrander [5] to orient reads to 5$^{\prime }\rightarrow 3^{\prime }$ and discard reads with aberrant primer configurations, producing oriented reads and unorientable reads (unknowns). Stage 3 scans the unknowns pool for internal adapters and recovers orientable sub-reads. Stage 4 scans oriented reads for adapter-associated structures and trims unsupported terminal residuals or splits supported internal concatemers. Outputs from Stages 3 to 4 are merged and aligned to the reference genome with minimap2 [10] in splice-aware mode. Stage 5 identifies homopolymer-mediated RT template-switching junctions from alignments and splits chimeras (Fig. 1b). DirectClean outputs cleaned FASTQ files and an interactive HTML report summarizing per-stage statistics (Supplementary Fig. S1).

**Figure 1.**
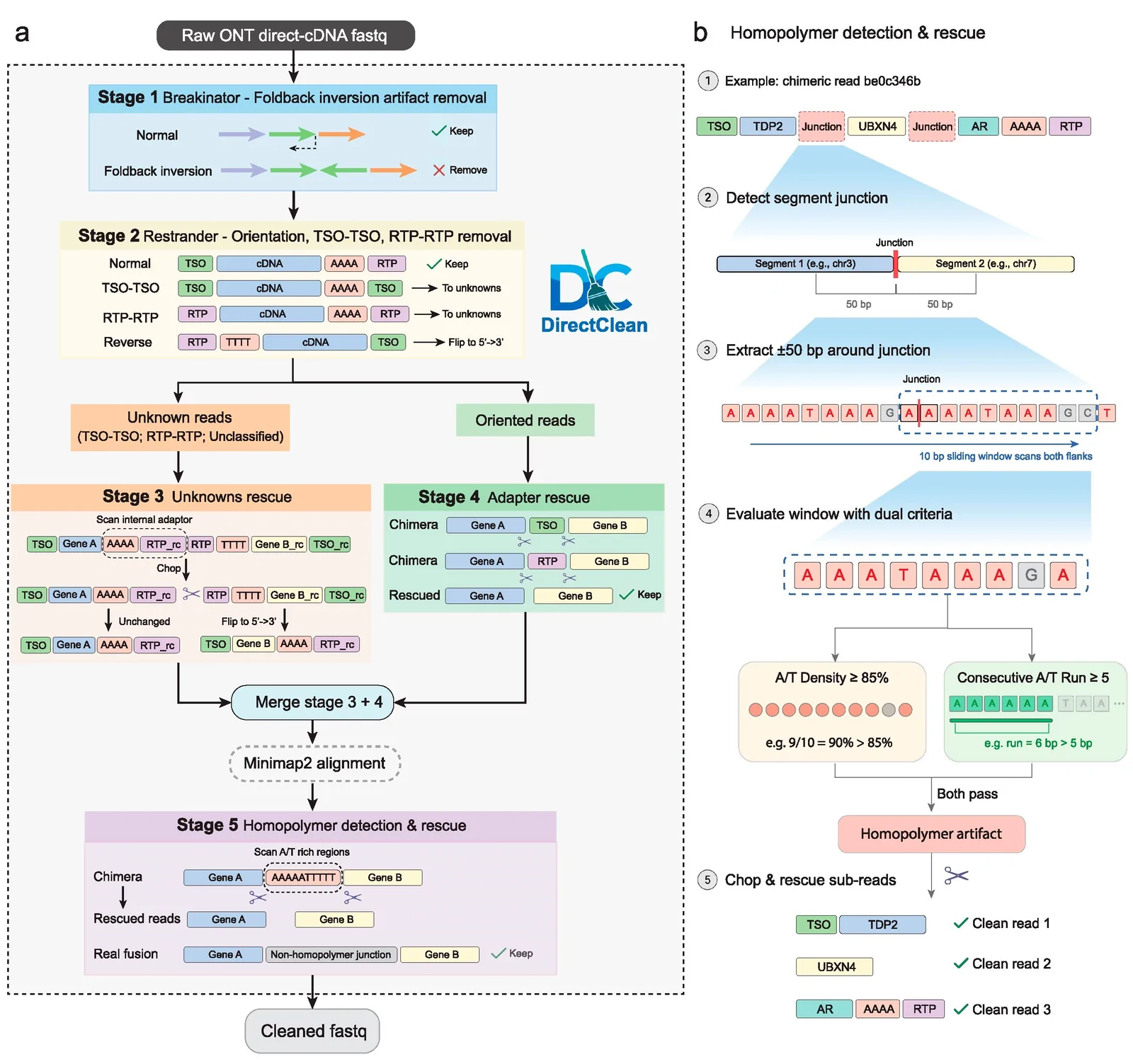
DirectClean pipeline and homopolymer artifact detection. (**a**) Workflow for ONT direct-cDNA preprocessing. DirectClean comprises five stages. Stage 1 (Breakinator) removes foldback inversions. Stage 2 (Restrander) orients reads to$5^{\prime }\rightarrow 3^{\prime }$ and assigns unclassifiable reads to an “unknowns” pool. Stage 2 outputs follow two paths: oriented reads proceed to Stage 4 (adapter rescue) to detect adapter-associated structures and trim terminal residuals or split internal concatemers; unknowns enter Stage 3 (unknowns rescue) for recovery via internal adapter detection. Outputs from Stages 3 to 4 are merged and aligned with minimap2. Stage 5 (homopolymer rescue) detects and splits chimeras at homopolymer-mediated RT template-switching junctions. Stages 3 to 5 recover usable sequence from supported artifact-associated structures by trimming or splitting reads and retaining qualifying segments. (**b**) Homopolymer detection, illustrated using chimeric read be0c346b from VCaP direct-cDNA data. Junctions are inferred from supplementary alignments, with 50-bp flanks extracted on each side. A 10-bp sliding window applies two criteria: A/T content *≥* 85% and longest consecutive A or T run *≥* 5 bp. Junctions meeting both are classified as RT template-switching artifacts and split into independent sub-reads.

### Internal adapter detection and sequence rescue

During direct-cDNA library preparation, incomplete RT or molecular ligation can produce chimeric reads containing internal TSO or RTP sequences. DirectClean resolves these concatemers via two modules: unknowns rescue (Stage 3) for reads that Restrander could not orient, and adapter rescue (Stage 4) for reads that Restrander successfully oriented. Both modules use edlib semi-global alignment [11] to detect adapter-associated signals, allowing up to three edits. Junction detection uses an anchor-and-extend search for internal poly(A), RTP reverse-complement (RTP_rc), and TSO signals, with at least two signals required. Internal searches exclude the first 100 bp and last 50 bp of each read to avoid expected terminal library structures.

Detected adapter-associated junctions are used to split reads into sub-reads. In Stage 4, a terminal segment lacking downstream TSO support and shorter than the preceding segment is instead trimmed as an adapter residual. Because Stage 3 processes reads that Restrander could not orient, the resulting sub-reads also lack directionality. Terminal poly(A)/poly(T) and TSO/RTP signals are used to assign 5$^{\prime }\rightarrow 3^{\prime }$ directionality, and reverse-strand segments are reverse-complemented. Segments that cannot be confidently oriented are discarded. Stage 4 skips this step as input reads are already oriented. For Stages 3 and 4, resulting segments shorter than 50 bp are discarded.

### Homopolymer artifact detection

Homopolymer-mediated template switching during RT generates chimeric reads that directly mimic genuine gene fusions at the alignment level. To detect these artifacts, Stage 5 scans the minimap2 BAM file for reads with supplementary alignments that map to different genomic loci. For each chimeric read, Stage 5 identifies the breakpoint position within the read sequence and extracts *±*50 bp of flanking sequence around the junction.

To distinguish RT artifacts from genuine gene fusions, a 10-bp sliding window scans the flanking region against two criteria (Fig. 1b): (i) A/T base density *≥*85%, and (ii) a consecutive single-nucleotide run of A or T *≥*5 bp. These criteria reflect the A/T-rich microhomology characteristic of RT template switching junctions [7, 8], with dual requirements preventing false flagging of dinucleotide repeats or short incidental A/T tracts. The numerical thresholds were selected empirically during initial method development by examining known artifact junctions and were fixed before the semi-synthetic benchmark was generated. Junctions meeting both criteria are classified as template-switching artifacts and split, with flanking sub-reads *≥*100 bp retained as independent sequences.

### Validation of rescue performance

We evaluated DirectClean on 5 348 910 ONT direct-cDNA reads from the VCaP prostate cancer cell line. An ablation study was first conducted to quantify the contribution of the sequence rescue modules by comparing downstream transcriptome metrics between Stages 1 and 2 only and the full five-stage pipeline. Reads were aligned to GRCh38 with minimap2 (splice-aware mode), transcript models were generated with IsoQuant v3.11.1 [12], and classified with SQANTI3 v5.4 [13] against GENCODE v41 [14]. Transcript support was defined as an IsoQuant count *> 0.001*. We defined two transcript sets: (i) rescue-associated transcripts, supported only after the full pipeline; and (ii) shared transcripts, supported after both Stage 1+2 and the full pipeline. We then compared homopolymer-rich coverage and RepeatMasker overlap between the two sets. Homopolymer-rich coverage was calculated using the DirectClean homopolymer criteria and compared with a two-sided Mann–Whitney *U* test; exon overlap with UCSC hg38 RepeatMasker elements [15, 16] was compared with a two-sided Fisher’s exact test.

For ground-truth validation, we generated 11 200 semi-synthetic reads from real VCaP sequence fragments: 600 adapter-associated constructs, 300 A/T-rich chimeras expected to be split, 300 non-qualifying chimeras expected to remain unsplit, and 10 000 untouched background reads (Fig. 2a). For each construct, we recorded whether DirectClean produced the expected outcome and how far the inferred breakpoint was from the designed junction.

**Figure 2.**
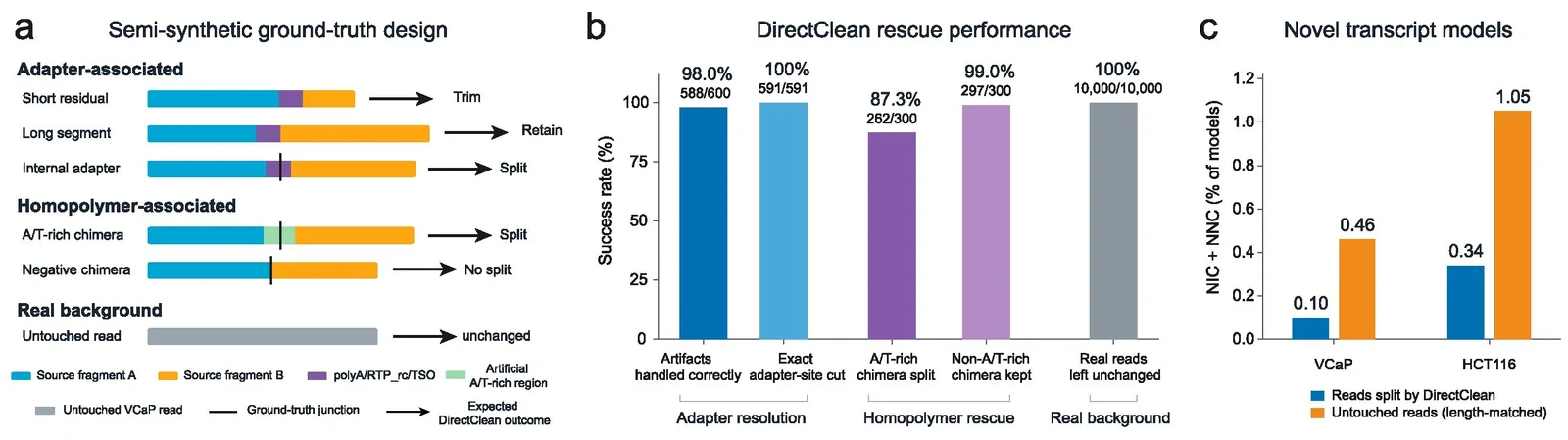
Semi-synthetic and real-data validation of DirectClean rescue performance. (**a**) Semi-synthetic ground-truth reads generated from VCaP sequences were designed to test adapter- and homopolymer-associated rescue, with non-qualifying chimeras and untouched reads included as negative controls. Arrows indicate the expected outcomes. (**b**) DirectClean performance on the semi-synthetic validation set, including adapter resolution, A/T-rich chimera splitting, retention of non-qualifying chimeras, and preservation of untouched reads. (**c**) NIC+NNC transcript models reconstructed from DirectClean split-derived reads and matched untouched reads in VCaP and HCT116.

As an independent real-data assessment, split-derived reads were compared with untouched controls in VCaP and HCT116. Controls were matched for primary-mapped read count and read-length distribution, and transcript models were reconstructed independently using the workflow described above. Novel-in-catalog (NIC) and novel-not-in-catalog (NNC) proportions were compared descriptively to assess whether splitting was associated with excess novel transcript structures.

### Benchmarking against existing tools

DirectClean and Pychopper were compared across four ONT direct-cDNA datasets: VCaP and three SG-NEx datasets (A549, HCT116, and HEYA8) [1]. Read yield, base retention, wall-clock runtime, and peak memory usage were compared; runtime benchmarks used 12 CPU cores. Pychopper v2.7.10 was run with the edlib backend and the default minimum mean read-quality threshold (-Q 7). A detailed VCaP comparison additionally included NanoPrePro v0.0.20 [9] (beta = 2.0).

We then compared chimeric read rates and gene fusion candidates across the raw, Pychopper-processed, and DirectClean-processed datasets. Chimeric reads were defined as mapped reads carrying an SA tag or supplementary alignment and were counted once per read name. Gene fusions were called by FusionSeeker with --minsupp 5 [17]. To assess artifact resolution, each chimeric read in the raw data was traced to the stage at which it was resolved or retained.

## Results

### DirectClean pipeline processing

We applied DirectClean to 5 348 910 raw ONT direct-cDNA reads from the VCaP prostate cancer cell line (Supplementary Table S1). Stage 1 removed 434 375 foldback inversion reads (8.1%). Stage 2 oriented the remaining reads and retained 3 386 185, while 1 528 350 were classified as unknowns. Stage 3 scanned the unknowns for internal adapter junctions and recovered 53 407 oriented reads. Stage 4 processed 231 974 oriented reads with adapter-associated structures and produced 233 921 retained rescued segments. After merging and alignment, Stage 5 detected 46 957 homopolymer-mediated chimeras and produced 95 332 retained segments (Supplementary Fig. S2). The final output contained 3 489 914 reads (65.2% retention).

### Contribution and validation of chop and rescue modules

To quantify the contribution of rescue modules (Stages 3–5), we compared transcriptome metrics between Stage 1 and 2 only and the full pipeline (Supplementary Table S2). The full pipeline produced 103 729 additional reads. It supported 29 343 transcripts, including 429 rescue-associated transcripts that were not supported after Stage 1–2. Read depth increased for 6435 transcripts.

The 429 rescue-associated transcripts had higher homopolymer-rich coverage than transcripts supported in both analyses (median 2.398% versus 1.474%; $P=4.32\times 10^{-10}$; Supplementary Table S3). RepeatMasker overlap was not enriched among rescue-associated transcripts.

Semi-synthetic ground-truth testing further evaluated rescue accuracy (Fig. 2b). DirectClean produced the expected trimming or splitting outcome for 98.0% of semi-synthetic reads containing adapter-associated artifacts, and all evaluable breakpoints were recovered at the designed adapter site. Overall, 87.3% of A/T-rich chimeric reads were split end-to-end as designed. For the subset processed by Stage 5, 95.3% of A/T-rich chimeras were correctly split, while 297/300 = 99.0% of non-qualifying chimeras were correctly left unsplit. None of the 10 000 untouched background reads were split.

In VCaP and HCT116, we further compared split-derived reads with matched untouched reads (Fig. 2c and Supplementary Table S4). NIC+NNC models accounted for 0.10% of models reconstructed from split-derived VCaP reads and 0.46% from matched untouched VCaP reads. In HCT116, the corresponding proportions were 0.34% and 1.05%. Thus, split-derived reads showed no enrichment of NIC/NNC transcript models relative to matched untouched reads.

### Comparison with existing preprocessing tools

We first compared DirectClean with Pychopper across four ONT direct-cDNA datasets (Table 1). DirectClean retained more reads and bases than Pychopper in all four datasets. Record yields ranged from 65.2% to 73.5% for DirectClean and from 51.4% to 65.9% for Pychopper. Base retention ranged from 65.4% to 73.3% for DirectClean and from 42.8% to 58.0% for Pychopper.

**Table 1.** Read and base yields of DirectClean and Pychopper across four ONT direct-cDNA datasets

			Pychopper			DirectClean		
Dataset	Data source	Input records	Output records	Record yield (%)	Base retention (%)	Output records	Record yield (%)	Base retention (%)
A549	SG-NEx	1 158 921	744 006	64.2	56.4	845 498	73.0	70.3
VCaP	This study	5 348 910	3 082 960	57.6	49.5	3 489 914	65.2	65.4
HCT116	SG-NEx	6 952 609	4 581 551	65.9	58.0	5 113 290	73.5	73.3
HEYA8	SG-NEx	10 799 478	5 549 278	51.4	42.8	7 683 480	71.1	69.6

Record yield and base retention are reported as percentages of the corresponding input records and bases.

A detailed comparison on VCaP additionally included NanoPrePro (Supplementary Table S5). DirectClean retained 3 489 914 reads (65.2%), compared to 3 082 960 (57.6%) for Pychopper. NanoPrePro retained only 84 159 reads (1.6%) and was excluded from downstream comparisons due to low yield.

DirectClean rescued 287 328 adapter-derived segments across Stages 3 and 4, versus 103 388 for Pychopper. It additionally removed 434 375 foldback inversions and resolved 46 957 homopolymer-mediated chimeras, artifact classes not targeted by Pychopper. Applying the DirectClean homopolymer detector to Pychopper output identified 70 140 residual homopolymer-associated artifacts, whereas none were detected in DirectClean output.

At the transcriptome level, DirectClean detected more FSM isoforms than Pychopper (20 535 versus 17 873; Supplementary Table S5). Transcripts with read support increased from 27 851 to 29 343. DirectClean provided read support for 6826 transcripts that were not supported after Pychopper processing. It also increased read depth for 16 077 transcripts.

DirectClean required 1.13–3.78 times the Pychopper wall-clock time across the four datasets. Peak memory usage was 19.65–24.00 GiB for DirectClean and 0.59–0.74 GiB for Pychopper (Supplementary Table S6).

### Chimeric read reduction and read fate analysis

To assess the effect of preprocessing on chimeric reads and downstream fusion detection, we compared chimeric read rates and fusion candidates across raw, Pychopper-processed, and DirectClean-processed data (Supplementary Table S7). Chimeric read rates decreased from 30.9% (raw) to 26.8% (Pychopper) and 16.9% (DirectClean). FusionSeeker with --minsupp 5 reported 1154 fusion candidates in the raw data, 1006 after Pychopper processing, and 878 after DirectClean processing.

To determine the fate of chimeric reads during DirectClean processing, we traced all 1 333 230 chimeric reads from the raw data through the pipeline (Supplementary Table S8). Overall, 69.1% were removed or resolved during processing: 32.6% by foldback removal, 27.5% by discarding unorientable reads, 5.9% by adapter rescue, and 3.1% by homopolymer rescue. The remaining 30.9% were retained in the final output as chimeric alignments.

## Conclusion

DirectClean provides an end-to-end preprocessing framework for ONT direct-cDNA data that integrates strand orientation, artifact removal, and sequence rescue. By combining established tools with three rescue modules (unknowns, adapter, and homopolymer rescue), it resolves chimeric reads via junction splitting rather than full-read removal, maximizing data retention in PCR-free protocols. Across four direct-cDNA datasets, DirectClean retained more reads and bases than Pychopper; in VCaP, it also supported additional transcripts and reduced the chimeric read rate from 30.9% to 16.9%. Semi-synthetic validation demonstrated accurate rescue of adapter-associated and A/T-rich chimeric reads. Split-derived reads showed no enrichment of NIC/NNC transcript models relative to matched untouched reads, indicating no excess of novel transcript structures after splitting. A current limitation is that homopolymer rescue relies on alignment-based detection, which may miss artifacts lacking supplementary alignments. Future work could incorporate sequence- or signal-level approaches to address this gap.

## Supplementary Material

lqag132_Supplemental_File

## Data Availability

The VCaP direct-cDNA sequencing data used in this study are available in the Gene Expression Omnibus (GEO) under accession number GSE277934. The publicly available SG-NEx A549, HCT116, and HEYA8 direct-cDNA datasets used for benchmarking are available in the European Nucleotide Archive (ENA) under accession number PRJEB44348 [1]. DirectClean is freely available at https://github.com/ylab-hi/DirectClean under the MIT license. The current release (v1.1.2) is also available through Bioconda and PyPI. A versioned release of DirectClean is archived in Zenodo under DOI: 10.5281/zenodo.20418867.
