# Supplementary material for: DirectClean: artifact-aware preprocessing and read rescue for Oxford Nanopore direct-cDNA transcriptome sequencing
Source: NAR Genom Bioinform. 2026 Oct 31;8(4):lqag132. doi: 10.1093/nargab/lqag132 (PMC13722937; doi:10.1093/nargab/lqag132)
Supplement: Supplementary file 1 [file lqag132_supplemental_file.pdf]

Supplementary Data

DirectClean: artifact-aware preprocessing and read rescue for Oxford Nanopore direct-cDNA transcriptome sequencing

Qingxiang Guo[1,*], Yangyang Li[1], and Rendong Yang[1,2,*]

[1]Department of Urology, Northwestern University Feinberg School of Medicine, 303 E Superior St, Chicago, IL 60611, USA
[2]Robert H. Lurie Comprehensive Cancer Center, Northwestern University Feinberg School of Medicine, 303 E Superior St, Chicago, IL 60611, USA

[*]To whom correspondence should be addressed: rendong.yang@northwestern.edu; qingxiang.guo@northwestern.edu

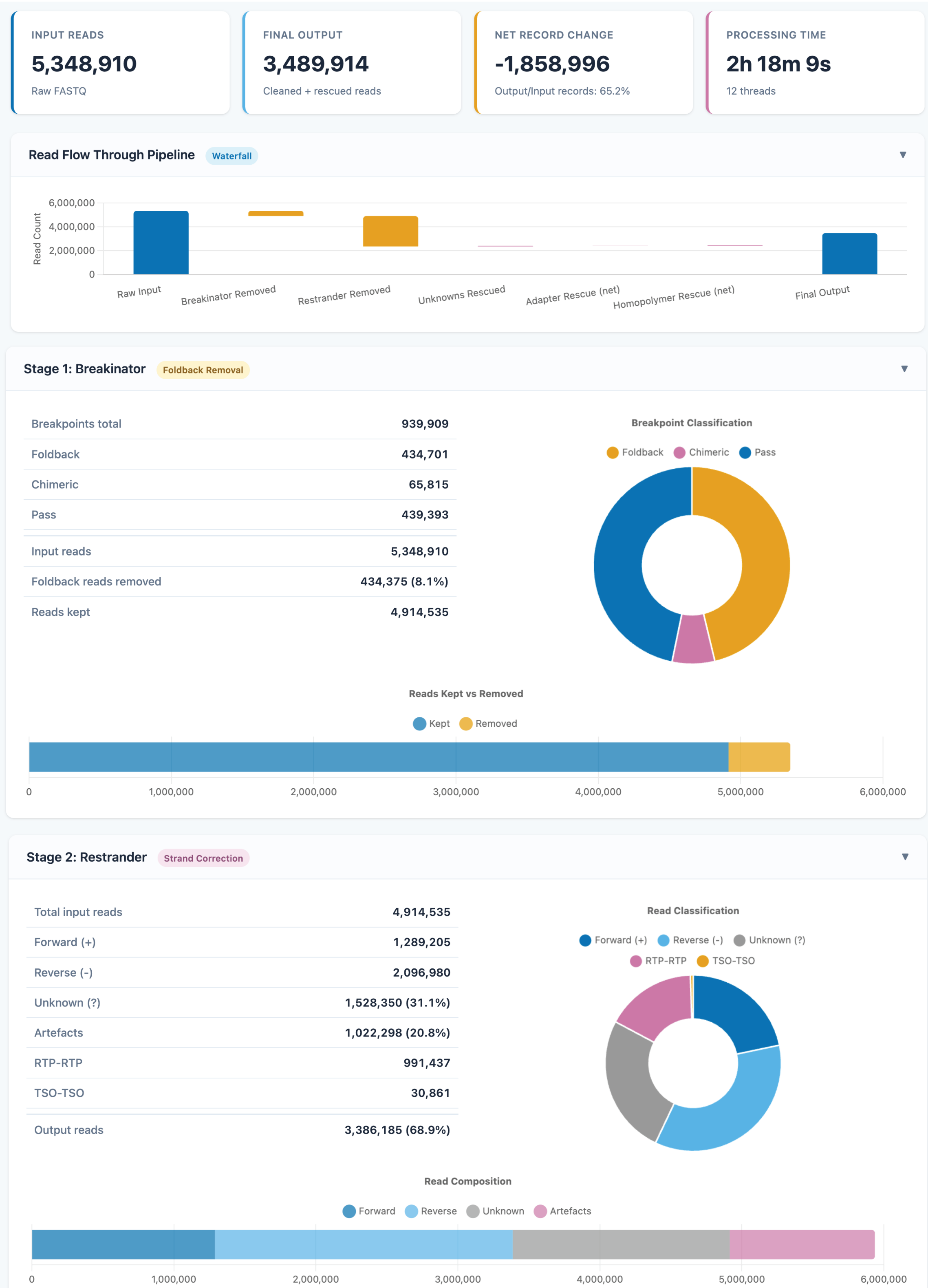

Supplementary Figure S1. Example HTML report generated by DirectClean. DirectClean produces a self-contained interactive HTML report summarizing pipeline statistics, including executive summary cards, a waterfall chart of read flow through each stage, and per-stage detail panels with classification breakdowns. The report shown was generated from the VCaP direct-cDNA dataset (5,348,910 input reads).

Alt text: DirectClean HTML report showing VCaP summary statistics, pipeline read flow, and per-stage read classifications and processing results.

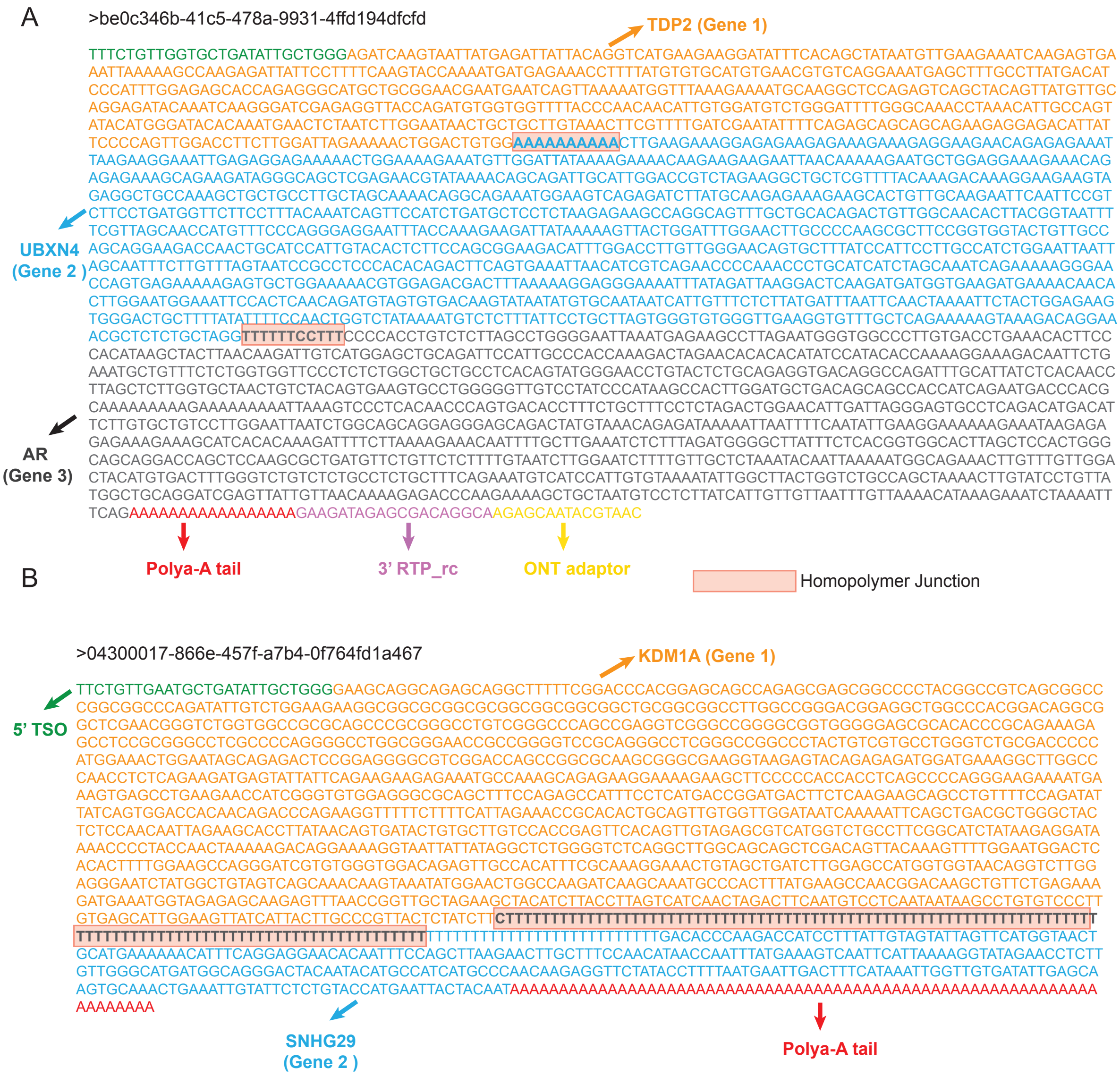

Supplementary Figure S2. Representative homopolymer-mediated RT template switching artifacts in VCaP direct-cDNA data. (A) Read be0c346b contains three gene segments (TDP2, UBXN4, AR) joined by two homopolymer junctions (shaded boxes). DirectClean Stage 5 detects both A/T-rich junctions and chops the read into three independent sub-reads. The 5' TSO, 3' polyA tail, RTP reverse complement, and ONT adaptor sequences are annotated. (B) Read 04300017 contains two gene segments (KDM1A, SNHG29) joined by an extended polyT tract (shaded box). DirectClean Stage 5 detects the junction and chops the read into two sub-reads.

Alt text: Two examples of homopolymer-mediated chimeric reads. DirectClean identifies A/T-rich junctions between gene segments and splits the reads into separate rescued sub-reads.

Supplementary Table S1. DirectClean pipeline summary on VCaP direct-cDNA data. Read counts are shown for each processing stage. Stages 1–2 perform foldback removal and strand orientation, and Stages 3–5 recover usable sequence from unclassified or chimeric reads. Stage 3 processes the unknown reads from Stage 2, and Stage 4 processes oriented reads. The Stage 5 input comprises the merged outputs of Stages 3 and 4; its reads-out count includes unchanged reads and retained segments generated by splitting. Thus, reads out can exceed reads in when one parent read yields multiple retained sub-reads.

Stage	Reads in	Reads out	Description
Raw input	5,348,910	—	ONT direct-cDNA input
1. Breakinator	5,348,910	4,914,535	Removed 434,375 foldback reads (8.1%)
2. Restrander	4,914,535	3,386,185	Oriented reads; 1,528,350 classified as unknowns
3. Unknowns rescue	1,528,350	53,407	Rescued from Stage 2 unknowns
4. Adapter rescue	3,386,185	3,388,132	231,974 parents processed; 233,921 retained rescued segments
5. Homopolymer rescue	3,441,539	3,489,914	46,957 parents chopped; 95,332 retained rescued segments
Final output	—	3,489,914	Cleaned reads for downstream analysis

Supplementary Table S2. Transcriptome quality metrics before and after DirectClean rescue modules. Comparison of Stage 1+2 only (Breakinator + Restrander) with the full DirectClean pipeline on VCaP direct-cDNA data. For supported-transcript comparisons, shared transcripts were detected in both analyses, whereas analysis-specific transcripts were detected only in the indicated analysis.

Metric	Stage 1+2 only	Full pipeline
Total output reads	3,386,185	3,489,914
FSM isoforms	20,449	20,535
ISM isoforms	830	836
NIC isoforms	1,369	1,375
NNC isoforms	742	757
Transcripts with read support	28,964	29,343
Shared supported transcripts	28,914	28,914
Analysis-specific supported transcripts	50	429
Net reads gained vs. Stage 1+2	—	+103,729
Net supported transcripts gained vs. Stage 1+2	—	+379
Transcripts with increased depth vs. Stage 1+2	—	+6,435

Supplementary Table S3. Sequence-context features of rescue-associated newly detected transcripts. Transcripts detected only after the full DirectClean pipeline ($n = 429$) were compared with transcripts detected in both Stage 1+2 and the full pipeline ($n =$ 28,914). Homopolymer-rich coverage is the fraction of transcript exonic sequence overlapping homopolymer-rich regions. RepeatMasker overlap indicates transcripts whose exons overlap an annotated RepeatMasker element.

Feature	Newly detected transcripts ($n = 429$)	Shared transcripts ($n = 28{,}914$)	P value
Homopolymer-rich coverage, median (IQR)	2.398% (0.702–4.016%)	1.474% (0.000–3.079%)	4.32×10^{-10}
Exonic RepeatMasker overlap, n/N (%)	267/429 (62.24%)	19,652/28,914 (67.97%)	0.0124

Supplementary Table S4. SQANTI3 classification of transcript models reconstructed from split-derived and matched untouched reads in VCaP and HCT116 direct-cDNA datasets. Split-derived reads were compared with matched untouched reads using equal numbers of primary-mapped reads within each dataset. Percentages are relative to the total number of transcript models in each group.

Dataset	Read group	Matched primary-mapped reads	Transcript models	FSM, n (%)	ISM, n (%)	NIC, n (%)	NNC, n (%)	NIC + NNC, n (%)	Other, n (%)
VCaP	Split-derived	76,950	3,062	3,042 (99.35)	16 (0.52)	2 (0.07)	1 (0.03)	3 (0.10)	1 (0.03)
VCaP	Matched untouched	76,950	6,049	5,993 (99.07)	25 (0.41)	23 (0.38)	5 (0.08)	28 (0.46)	3 (0.05)
HCT116	Split-derived	180,942	6,459	6,389 (98.92)	45 (0.70)	16 (0.25)	6 (0.09)	22 (0.34)	3 (0.05)
HCT116	Matched untouched	180,942	8,917	8,699 (97.56)	119 (1.33)	65 (0.73)	29 (0.33)	94 (1.05)	5 (0.06)

Supplementary Table S5. Benchmarking on VCaP direct-cDNA data. Comparison of read yield, artifact resolution, and transcriptome metrics across Pychopper, NanoPrePro, and DirectClean using the same input dataset (5,348,910 reads). NanoPrePro transcriptome metrics are not reported because of its low read retention ($< 2\%$).

Metric	Pychopper	NanoPrePro	DirectClean
**Read processing and yield**			
Clean output reads	3,082,960	84,159	3,489,914
Retention rate	57.6%	1.6%	65.2%
**Artifact resolution**			
Adapter-rescued segments	103,388	0	287,328
Foldback reads removed	—	—	434,375
Homopolymer artifact reads chopped	—	—	46,957
Residual homopolymer artifacts	70,140	—	0
**Downstream transcriptome impact**			
FSM isoforms (SQANTI3)	17,873	—	20,535
ISM isoforms (SQANTI3)	414	—	836
Transcripts with read support	27,851	—	29,343
Net reads gained vs. Pychopper	—	—	+406,954
Newly detected transcripts vs. Pychopper	—	—	+6,826
Transcripts with increased depth vs. Pychopper	—	—	+16,077

Supplementary Table S6. Wall-clock runtime and peak memory usage of DirectClean and Pychopper across four ONT direct-cDNA datasets using 12 CPU cores. Runtime ratios were calculated as DirectClean wall-clock time divided by Pychopper wall-clock time.

Dataset	Input records	Input bases (Gb)	Wall-clock time		Runtime ratio	Peak RAM (GiB)	
			Pychopper	DirectClean		Pychopper	DirectClean
A549	1,158,921	1.27	00:16:37	00:18:42	1.13	0.61	19.65
VCaP	5,348,910	7.17	01:03:24	02:25:35	2.30	0.74	20.51
HCT116	6,952,609	8.98	01:08:38	03:45:37	3.29	0.59	19.74
HEYA8	10,799,478	11.61	01:12:50	04:35:38	3.78	0.59	24.00

Supplementary Table S7. Chimeric read rates and gene fusion candidates across preprocessing pipelines. Raw, Pychopper-processed, and DirectClean-processed reads were compared using the same VCaP direct-cDNA input (5,348,910 reads). Gene fusion candidates were identified using FusionSeeker with `--minsupp 5`.

Metric	Raw	Pychopper	DirectClean
Chimeric rate	30.9%	26.8%	16.9%
Chimeric reads	1,333,230	770,158	480,439
Fusion candidates	1,154	1,006	878

Supplementary Table S8. Classification of raw chimeric reads by DirectClean processing stage. Each of the 1,333,230 chimeric reads in the unprocessed data was tracked through the pipeline to determine whether it was removed, resolved, or retained.

Fate	Reads	Percent
Removed or resolved during processing	921,485	69.1%
Stage 1: Foldback removed	434,375	32.6%
Stage 2: Unknowns discarded	367,116	27.5%
Stage 4: Adapter chopped	78,976	5.9%
Stage 5: Homopolymer chopped	41,018	3.1%
Retained as chimeric alignments	411,745	30.9%
Total	1,333,230	100.0%
